# Intensity-modulated radiotherapy for squamous cell carcinoma of the anal canal: Efficacy of a low daily dose to clinically negative regions

**DOI:** 10.1186/1748-717X-6-134

**Published:** 2011-10-06

**Authors:** Jason A Call, Michael G Haddock, J Fernando Quevedo, David W Larson, Robert C Miller

**Affiliations:** 1 Department of Radiation Oncology, Mayo Clinic, 200 First St SW, Rochester, MN 55905, USA; 2 Division of Medical Oncology, Mayo Clinic, 200 First St SW, Rochester, MN 55905, USA; 3 Division of Colon and Rectal Surgery, Mayo Clinic, 200 First St SW, Rochester, MN 55905, USA

**Keywords:** anal cancer, chemotherapy, intensity-modulated radiotherapy, squamous cell carcinoma

## Abstract

**Background:**

We aimed to assess outcomes of patients with anal cancer who underwent intensity-modulated radiotherapy (IMRT) and received less than 1.80 Gy/day.

**Methods:**

We retrospectively reviewed our experience using a low fractional dose (< 1.80 Gy) of IMRT to elective nodal areas for patients receiving chemoradiotherapy for anal cancer. Three-year freedom from any disease relapse and overall survival were estimated using Kaplan-Meier curves. We documented the daily dose that was delivered to clinically uninvolved regions and to areas of gross disease. Incidence of regional failures in high (≥ 1.80 Gy) and low (< 1.80 Gy) daily dose regions was assessed.

**Results:**

Thirty-four consecutive patients (median age, 59 years) received IMRT from June 2005 through January 2009. Median follow-up duration was 22 months. Twenty-eight patients had T1 or T2 disease and 6 had T3 or T4 disease. Fourteen patients had nodal metastases. Median treatment dose was 50.40 Gy (range, 48.60-57.60 Gy) in 25 to 32 fractions. The range of fractional doses to clinically negative volumes was 1.28 to 1.80 Gy. Seventeen patients (50%) received a fractional dose of less than 1.60 Gy, 13 (38%) received less than 1.50 Gy, and 9 (26%) received less than 1.40 Gy to at least a portion of the clinically negative volume. Three-year freedom from relapse was 80%, and 3-year overall survival was 87%. No patient had treatment failure in the clinically negative volume that received a low daily dose.

**Conclusions:**

Our data support using doses between 1.50 and 1.80 Gy/day to clinically uninvolved regions.

## Introduction

Fluorouracil (FU) and mitomycin C (MMC) combined with radiotherapy is the standard treatment for squamous cell carcinoma arising in the anal canal (1-4). Trials of conventional radiotherapy techniques have shown significant toxicity (1), and interest has focused on intensity-modulated radiotherapy (IMRT) in this setting, with the hope of decreasing severe toxicity. The Radiation Therapy Oncology Group (RTOG) has developed an IMRT protocol that has been tested in a multi-institutional study (5). The protocol uses a daily fraction dose that varies according to the specific target volume. Such a technique allows gross disease and elective areas to receive different total doses while the number of fractions remains the same. Under certain conditions, elective regions could receive a fractional dose as low as 1.50 Gy per day. Historically, anal cancer has been treated with doses of at least 1.80 Gy per day, with a shrinking-field technique over the course of treatment. Data on doses less than 1.80 Gy per day are lacking. At our institution, use of such lower doses in the treatment of anal cancer is common when using IMRT. This study was undertaken to review our experience of low-dose IMRT (< 1.80 Gy per day) in the treatment of anal cancer and to determine the rate of regional failures with this treatment regimen.

## Methods and Materials

This study was approved by the Mayo Clinic Institutional Review Board. We reviewed all patients with squamous cell carcinoma of the anus who received chemoradiotherapy with IMRT from June 2005 through January 2009 at Mayo Clinic, Rochester, Minnesota. All living patients authorized review of their medical record in accordance with Minnesota state law. Data on patient and tumor characteristics, details about radiotherapy, and outcomes of disease control and survival were obtained from the medical record. All cancers were evaluated (assigned a TNM stage) according to the American Joint Committee on Cancer Staging Manual, seventh edition (6). Regional failures were recorded, along with the dose received during IMRT. Follow-up primarily consisted of a physical examination, with imaging studies performed at the discretion of the supervising physician. Biopsies were not routinely performed if physical examination findings were favorable.

### Statistical Analysis

The Kaplan-Meier method was used to calculate and estimate rates of overall survival and freedom from any disease relapse. Data were analyzed using JMP software (version 8.0; SAS Institute, Cary, North Carolina).

## Results

### Patient and Tumor Characteristics

We identified 34 consecutively treated patients who received definitive IMRT and chemotherapy (FU alone [n = 1] or a combination of FU and MMC [n = 33]). Patient characteristics are displayed in Table 1. Median age was 59 years. Twenty-eight patients (82%) had T1 or T2 disease, and 6 (18%) had T3 or T4 disease. Fourteen patients (41%) had nodal disease. The median duration of follow-up was 22 months.

**Table 1:** Patient Characteristics (N = 34)

Characteristic	Value
Age, median (range), y	59 (46-85)
IMRT dose, median (range), Gy	50.40 (48.60-57.60)
Chemotherapy, No.	
FU with MMC	33
FU only	1
TNM category, No.	
T1	10
T2	18
T3	4
T4	2
N0	20
N1	7
N2	3
N3	4
M0	34
	

Abbreviations: FU, fluorouracil; IMRT, intensity-

modulated radiotherapy; MMC, mitomycin C.

### Radiotherapy

Details of radiotherapy are shown in additional file 1 Tables 2 and 3, stratified by nodal disease status (stage N0 vs N+ disease). Radiation was delivered with 6-MV photon beams to 11 fields (n = 1) or to 9 fields (n = 33). It was common to treat the upper pelvis with a lower dose than the lower pelvis. (Upper and lower pelvis volumes typically were delineated around the bottom of the sacroiliac joints.) The treating clinician individualized gross tumor volumes, clinical target volumes, and planning target volume expansions for each patient. A simultaneous integrated boost (SIB) technique was used to treat the gross tumor volume and elective areas in a single treatment plan (ie, applying different doses per fraction to different target volumes). Use of low fractional doses of radiation was common. Doses ranged from 48.60 to 57.60 Gy (median, 50.40 Gy) in 25 to 32 fractions.

Gross disease was treated with a daily fraction of 1.80 to 2.25 Gy. The gross tumor volume was commonly treated with a margin that varied according to the discretion of the treating clinician but would often include immediately adjacent lymph node tissues. Doses to electively covered areas that were outside those margins are specified in additional file 1 Tables 2 and 3.

The range of doses to clinically negative volumes was 1.28 to 1.80 Gy per day. All patients received less than 1.80 Gy per fraction to some portion of the electively covered volume. Seventeen patients (50%) received a fractional dose less than 1.60 Gy, 13 (38%) received less than 1.50 Gy, and 9 (26%) received less than 1.40 Gy to at least a portion of the clinically negative volume. Positive nodes received a median fractional dose of 1.93 Gy (range, 1.80-2.25 Gy).

### Disease Control and Overall Survival

**Figure 1:**
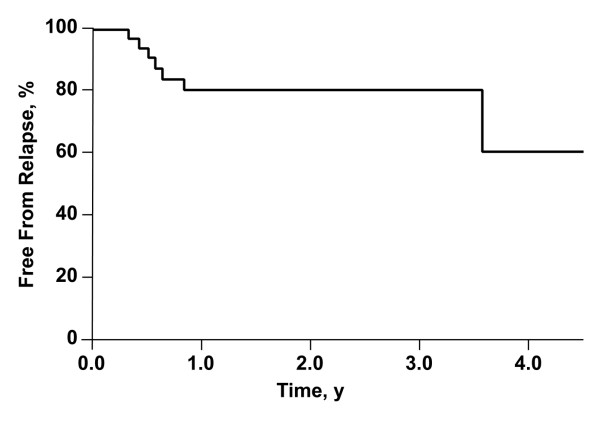
**Estimated Rate of Freedom From Any Disease Relapse in All Patients**. The rate at 3 years was 80%.

The 3-year freedom from any disease relapse was 80% (Figure 1). Three patients had a local failure, one patient had a regional lymph node failure, and 4 had cancer recur at distant sites (one had a distal failure 3 months after a local failure). The patient with the regional failure had progression at a site of gross nodal disease that was treated with a dose of 56.25 Gy in 25 fractions (2.25 Gy per day). No treatment failures were observed in the target volumes that received less than 1.80 Gy per day (100% regional control in low-dose areas).

Three patients died during the follow-up period. One patient died of cardiac arrest 6 months after the diagnosis of anal cancer; the patient was disease free at the time of death. The second patient died of sepsis associated with metastatic anal cancer 10 months after diagnosis. The third patient was a 79-year-old woman with a history of congestive heart failure and chronic obstructive pulmonary disease; she died after 26 months of follow-up. Although the cause of death was not documented in this case, she did not have evidence of recurrent cancer during the follow-up period. The remaining 31 patients were alive at the time of manuscript preparation, with a median survival of 23 months. The estimated survival at 3 years was 87% for the entire group.

## Discussion

IMRT for anal cancer is currently under investigation in a multi-institutional study. Using IMRT with SIB to treat different targets with different daily doses often results in some areas receiving less than the conventional fractional doses of radiation (ie, < 1.80 Gy). This technique has some treatment benefits. Multiple IMRT plans could be used to allow no change in fractional dose during the treatment period, but this requires additional planning and quality assurance and also extends the treatment time. Increased treatment times may be associated with poor disease control (7-13). Use of IMRT to deliver an SIB has the advantage of being able to deliver the radiation in a shorter time. However, this necessitates varying the fractional dose, and thus clinically negative areas may be treated with lower daily doses than what has typically been administered in anal cancer clinical trials. Data on the biologic effects and clinical outcomes of such low doses are lacking.

Historically, anal cancers were treated with surgical therapy involving an abdominoperineal resection. Interest in improving outcomes for these patients led to the discovery that these tumors responded to chemotherapy and radiotherapy. Such therapy, delivered in a neoadjuvant fashion, decreased the failure rate compared with that of surgery alone (14,15). This ultimately led to a primary approach of chemoradiotherapy, obviating the need for surgery for patients with a complete response and negative biopsy findings (16).

Radiotherapy and concurrent FU and MMC is the current standard of care and allows many patients to avoid having a colostomy. Several prospective trials on chemoradiotherapy have been performed in the study of this disease. Although there has been some variation in technique, an overview of these trials shows that they generally have used doses of at least 1.80 Gy per day. Phase 3 trials performed by the United Kingdom Coordinating Committee on Cancer Research (UKCCCR) and the European Organization for Research and Treatment of Cancer (EORTC) demonstrated that chemoradiotherapy with these agents was superior to radiotherapy alone in terms of local control and the ultimate need for a colostomy (2,4). In the UKCCCR trial, treatment involved a technique of opposed anterior and posterior fields to treat the central axis with a dose of 45 Gy in 20 to 25 fractions over 4 to 5 weeks. Patients with less than 50% response were treated surgically, and all others were recommended to receive a boost (15 Gy in 6 fractions, by electrons, photons, or an interstitial implant) over 2 to 3 days. In the EORTC trial, initial fields (3- or 4-field technique) were treated with 45 Gy (1.80 Gy per day) over 5 weeks. After a 6-week break, patients with a complete response then received a further boost of 15 Gy, whereas those with a partial response received 20 Gy. Other trials have examined the optimal chemotherapy to be delivered with radiotherapy in the definitive treatment of anal cancer. Continuous infusion of FU alone (1,000 mg/m^2 ^per day over 96 hours, starting days 1 and 29 of radiotherapy) was inferior to chemotherapy with MMC (10 mg/m^2 ^on days 1 and 29) in a phase 3 trial performed by the RTOG and Eastern Cooperative Oncology Group (trial RTOG 87-04/ECOG 1289) (3). The first 45 Gy of radiotherapy were concomitant with the first 2 cycles of chemotherapy and used parallel opposed fields and a daily fraction of 1.80 Gy. After 30.6 Gy was administered, the top field border was reduced from the interspace between L4 and L5 to the bottom of the sacroiliac joints. This field was continued until a dose of 36 Gy was administerd. Finally, a boost field to the tumor alone was used until a total dose of 45 Gy was achieved.

If a tumor was still palpable immediately after the initial 45 Gy, the patient had a boost treatment with another 5.4 Gy. For patients with N1 disease, both inguinal regions were initially treated with a dose of 50.40 Gy at a prescription depth of 3 cm. After 4 to 6 weeks, patients were assessed by a biopsy; if results were positive, they received further therapy. For patients with biopsy results showing residual primary disease, salvage therapy consisted of 9 Gy in 5 fractions (delivered with electrons or photons) and the same regimen of FU plus cisplatin (100 mg/m^2^) on day 2 of radiotherapy. Patients with palpable inguinal disease after administration of 45 to 50.4 Gy received an additional 9 Gy.

Trial RTOG 98-11 (1) attempted to substitute cisplatin for the MMC component of therapy. Patients were randomized to 1 of 2 treatment arms: 1) concurrent FU, MMC, and radiotherapy; or 2) neoadjuvant cisplatin and FU alone, followed by concurrent chemoradiotherapy with cisplatin and FU. The treatment arm with MMC and FU had a significantly reduced colostomy rate. The radiotherapy was also administered with shrinking fields; after 30.6 Gy was administered, the superior border was moved down from L5 and S1 to the bottom of the sacroiliac joints, and a minimum of 14.4 Gy of additional radiation was administered to the tumor (all at 1.80 Gy per day). Node-negative patients received 36 Gy to inguinal regions. Certain patients (stage T3 or T4, node positive, or N2 with residual disease) were treated with a boost of 10 to 14 Gy at 2 Gy per fraction, for a total tumor dose of 55 to 59 Gy.

A second phase 3 trial, conducted in the United Kingdom, examined outcomes after replacing MMC with cisplatin that was administered concurrently (no initial gap) with a radiotherapy dose of 50.4 Gy (17). This trial showed no significant improvement in complete response rate with concurrent cisplatin (95%) compared with MMC (94%), and the need for a colostomy was similar between groups. Currently, radiotherapy delivered concurrently with FU and MMC remains the standard of care for squamous cell carcinoma of the anal canal.

The RTOG initiated a multi-institutional effort to prospectively treat patients with IMRT-based chemoradiotherapy (RTOG 0529) (5). In this protocol, patients received IMRT with SIB to treat the elective areas and gross disease in the same number of fractions. IMRT was able to significantly reduce the grade 2+ dermatologic and grade 3+ gastrointestinal/genitourinary events compared with the results of the RTOG 98-11 trial. Fractional doses varied by the clinical situation but were as low as 1.5 Gy per day to clinically negative areas.

Our data indicate that a low dose per fraction when treating with an SIB technique may be effective for clinically negative areas. It was common to treat at least a portion of the elective areas with less than 1.80 Gy per day. We observed only one regional failure that occurred at the site of a grossly positive node that received a dose of 56.25 Gy (2.25 Gy per day). No patients in our series had treatment failure within the elective, low-dose volume. Kachnic et al (18) reported results from several centers in Boston using an IMRT technique that commonly treated elective nodal areas with doses as low as 1.5 Gy per fraction. With a median follow-up of 24 months, these authors noted a 2-year local control rate of 95%, and only 2 of 43 patients had a pelvic recurrence. In addition, trial RTOG-0529 used a similar technique for treating patients by using IMRT to deliver a low daily dose to elective areas. Preliminary 2-year results have been reported (19), and the locoregional failure rate at 2 years was 20%. Long-term results are not yet available to assess the effectiveness of the approach in a multi-institutional setting. The results presented here add to the growing body of data supporting the use of IMRT with SIB for anal cancer. Regional control was excellent, despite the common use of low doses per fraction.

Limitations of our data include the retrospective nature of this study. In addition, no standard method was used to prescribe radiotherapy. In addition, the relatively small size (34 patients) and short follow-up (22 months) in this report should be noted. It was common for patients to receive a low fractional dose to at least a portion of the elective volume; however, specific dosages to certain volumes were individualized according to the judgment of each radiation oncologist. All patients in this study received concurrent chemotherapy. We do not know whether the same low rate of regional failure would have been observed if such radiosensitizing agents were not used.

In conclusion, our results indicate that low fractional doses of radiation may be appropriate when using IMRT for squamous cell carcinoma of the anus along with concurrent chemotherapy. A daily dose between 1.50 and 1.80 Gy per day to clinically negative areas, prescribed according to the RTOG technique, may be appropriate in certain clinical situations. No treatment failures were noted in the low-dose prescription volumes, despite the frequent use of fractional doses less than 1.80 Gy.

## Authors' contributions

JAC: Reviewed charts, gathered data, and drafted the manuscript. MGH: Aided in the design of the study, helped draft the manuscript. DWL: Aided in the design of the study, helped draft the manuscript. JFQ: Aided in the design of the study, helped draft the manuscript. RCM: Participated in study design and coordination and helped draft the manuscript. All authors read and approved the final version of this manuscript.

## Conflicts of interest

The authors declare that they have no competing interests.

## Abbreviations

EORTC: European Organization for Research and Treatment of Cancer; FU: fluorouracil; IMRT: intensity-modulated radiotherapy; MMC: mitomycin C; RTOG: Radiation Therapy Oncology Group; SIB: simultaneous integrated boost; UKCCCR: United Kingdom Coordinating Committee on Cancer Research
